# Analytical purification of a 60-kDa target protein of artemisinin detected in *Trypanosoma brucei brucei*

**DOI:** 10.1016/j.dib.2015.09.026

**Published:** 2015-10-03

**Authors:** Benetode Konziase

**Affiliations:** Graduate School of Pharmaceutical Sciences, Osaka University, 1-6 Yamada-oka, Suita, Osaka 565-0871, Japan

**Keywords:** ddH_2_O, double-distilled water, MeOH, methanol, SDS-PAGE, sodium dodecyl sulfate polyacrylamide gel electrophoresis, Micropurification, Protein, Purity, Reverse staining, Gel, Electrophoresis

## Abstract

Here we describe the isolation and purity determination of *Trypanosoma brucei (T. b.) brucei* candidate target proteins of artemisinin. The candidate target proteins were detected and purified from their biological source (*T. b. brucei* lysate) using the diazirine-free biotinylated probe **5** for an affinity binding to a streptavidin-tagged resin and, subsequently, the labeled target proteins were purified by sodium dodecyl sulfate-polyacrylamide gel electrophoresis (SDS-PAGE). We herein showed the electrophoresis gel and the immunoblotting film containing the 60-kDa trypanosomal candidate target protein of artemisinin as a single band, which was visualized on-gel by the reverse-staining method and on a Western blotting film by enhanced chemiluminescence. The data provided in this article are related to the original research article “Biotinylated probes of artemisinin with labeling affinity toward *Trypanosoma brucei brucei* target proteins”, by Konziase (Anal. Biochem., vol. 482, 2015, pp. 25–31. http://dx.doi.org/10.1016/j.ab.2015.04.020).

## Specifications table

**Table:** 

Subject area	Biochemistry
More specific subject area	Protein micropurification
Type of data	Experimental procedures, stained polyacrylamide gel, immunoblotting film
How data was acquired	Affinity labeling/binding, SDS-PAGE, gel reverse staining, Western blotting, gel excision, protein elution from gel matrix, protein concentration, ultrafiltration, ultracentrifugation: TOMY MX-300 (Tokyo Seiko)
Data format	Text, figures
Experimental factors	Parasite (*T. b. brucei*) lysate was prepared as the biological source of protein samples
Experimental features	The polyacrylamide gel remained wet or immersed in liquid as to avoid destructive air contact; only double distilled water or MilliQ water should be used
Data source location	Osaka, Japan
Data accessibility	Data are available with this article

## Value of the data

• To open the route to N-terminal sequencing of the *T. b. brucei* 60-kDa candidate target protein of artemisinin.
• To provide strategy for purification of trypanosomal target proteins of artemisinins.
• To prove the efficiency of the diazirine-free probe **5** as an affinity purification tool for pathogenic target proteins.
• Considering the proven efficiency *in vitro* of artemisinins against other tropical pathogens such as *Trypanosoma cruzi*, Leishmania [2] or Schistosoma [3], our data could provide other researchers the necessary molecular tool and method for micropurification of potential target proteins in these pathogens.

## Data

The data displayed here represent the outcome of micropurification steps and visualization techniques used for purifying *T. b. brucei* target proteins of artemisinin, which were previously detected at 60, 40 and 39 kDa by immunoblotting [1]. The polyacrylamide gel and immunoblotting film presented here below reflect, on the one hand, the successful isolation of the 60-kDa protein band but, on the other hand, the difficulty to isolate both low-abundance proteins at 40 and 39-kDa. It should be noted that a two-dimensional SDS-PAGE for further purity assessment of the 60-kDa target protein band was not yet performed.

## 1 Materials and methods

### 1.1 *T. b. brucei* lysate preparation protocol

The *Trypanosoma brucei brucei* lysate was prepared as described in the associated research article [1]. The trypanosome lysis buffer used was prepared by mixing 9.99 mL of phosphate buffered saline (PBS), 10 μL of Triton-X 100 (0.1%), 88.54 mg of NaCl (150 mM), and 100 μL of protease inhibitor cocktail (1%).

### 1.2 Isolation of candidate target proteins of artemisinin

**Fig. 1:**
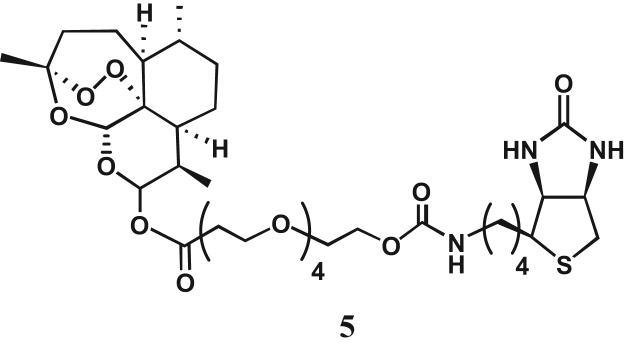
Biotinylated probe **5** used for affinity labeling and purification of the candidate target proteins of artemisinin from the *T. b. brucei* lysate.

In an Eppendorf tube, 100 μM of probe **5** (Fig. 1) that was previously synthesized as described in [4] was inoculated directly into the parasite lysate and the whole preparation was incubated at 37 °C in a 5% CO_2_ atmosphere incubator for 5 min.^3^ Then, streptavidin-tagged resins were added to immobilize labeled proteins during an overnight rotation (~15-h, 4 °C). The supernatant was removed and the resins were washed twice with trypanosome lysis buffer, and then Laemmli׳s sample loading buffer (20 μL) was added and mixed well with the resins by pipetting. The labeled proteins were unbound from the resins in the Laemmli׳s sample loading buffer with a heat treatment (95 °C, 5 min). The protein samples (15 μL) were separated using sodium dodecyl sulfate-polyacrylamide gel electrophoresis (SDS-PAGE, 500 V, 20 mA, 65 min). Next, the electrophoresis gel was reverse stained as described in Section 1.3. The on-gel detected protein bands were excised, transferred in an Eppendorf tube, destained, and crushed in the Laemmli׳s SDS-PAGE running buffer. After elution by vigorous agitation, the filtered protein samples were concentrated by ultracentrifugation as described in Section 1.5, and then subjected to SDS-PAGE in duplicate. The first gel underwent usual Western blotting procedures and was visualized by enhanced chemiluminescence, whereas the second gel was reverse stained, thereby allowing on-gel detection.

### 1.3 Protein detection by reverse staining of the polyacrylamide gel

The reverse staining [5], [6], [7], [8] (or negative staining or imidazole–zinc staining) is a protein detection method using imidazole and zinc salts in electrophoresis gels. The principle of the method consists in selectively precipitating a white opaque imidazole–zinc complex in the electrophoresis gel except in the zones where protein bands are located, which zones remain transparent. As a procedure, the pre-treatment solution (10% aq. MeOH in ddH_2_O) was poured in a plastic tray. The polyacrylamide gel (obtained in Section 1.2) was immersed into the pre-treatment solution and the tray was shaken smoothly for 5 min. The gel was removed from the pre-treatment solution and immersed into 100 mL of fresh ddH_2_O in a separate plastic tray, which was shaken smoothly for 30 s. Next, the gel was immersed into the Staining solution R-1 (10 mL reverse-staining kit R-1 reagent (Bio-Rad) in 50 mL ddH_2_O) in a separate plastic tray that was shaken for 15 min (in the case of 5–20% gradient polyacrylamide gel). The gel was removed from the Staining solution R-1 and immersed into 100 mL of fresh ddH_2_O in a separate tray, which was shaken smoothly for 30 s. Later, the gel was immersed into the Development solution R-2 (10 mL reverse-staining kit R-2 reagent (Bio-Rad) in 50 mL ddH_2_O) in a separate plastic tray that was shaken for 1–3 min until protein bands were visualized. The gel was washed in 100 mL of fresh ddH_2_O for 2 min. The water was discarded and the gel was washed a second time with fresh ddH_2_O for 5 min.

### 1.4 Protein recovery from the electrophoresis gel

The reverse-stained gel was placed on a plastic wrap over a dark-colored background and the on-gel detected protein bands were excised with a sterile scalpel, and then transferred in an Eppendorf tube. Laemmli׳s SDS-PAGE running buffer (500 μL) was added and the Eppendorf tube was shaken gently for 10 min until destaining occurred. The supernatant was removed, 500 μL of Laemmli׳s SDS-PAGE running buffer was added again, and the Eppendorf tube was shaken gently for 10 min once more. The supernatant was discarded and 100 μL of Laemmli׳s SDS-PAGE running buffer was added. The immersed electrophoresis gel was manually crushed into tiny pieces with a clean spatula. Then, 100 μL of Laemmli׳s SDS-PAGE running buffer was added for achieving a final volume of 200 μL. The whole suspension was shaken vigorously for 1 h, transferred into a centrifugal filtration tube (ATTO AB-1171), and then centrifuged at 14,000*g* for 10 min at room temperature. The filtrate solution was stored at 4 °C.

### 1.5 Protein concentration by ultracentrifugation

The protein filtrate (obtained in Section 1.4) was transferred into a molecular weight-filter tube (YM-10 Microcon). The volume was adjusted up to 500 μL with Laemmli׳s SDS-PAGE running buffer and the whole preparation was centrifuged at 14,000*g* at room temperature for 40 min. The retentate of *ca.* 10 μL was recovered from the retention membrane as the concentrated protein suspension. The sample reservoir was placed upside down in a new vial and centrifuged for 3 min at 1000*g* for transferring protein retentate to the vial. Finally, the concentrated protein suspension was analyzed by SDS-PAGE or by Western blotting.

## 2 Results

### 2.1 Isolation of *T. b. brucei* candidate target protein of artemisinin

**Fig. 2:**
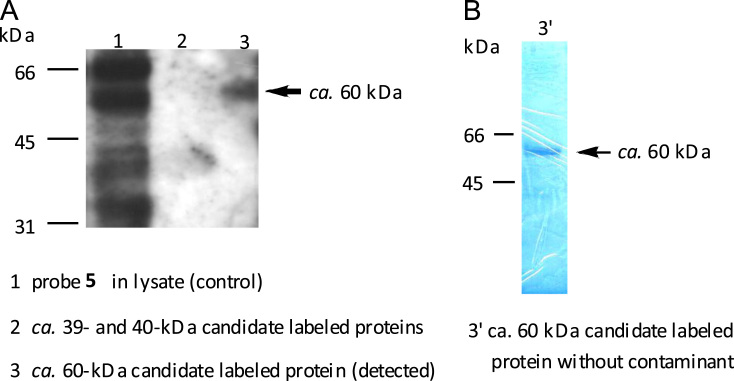
Isolation and purity determination of candidate target proteins of artemisinin by the reverse-staining method. Gel-excised candidate protein bands were visualized by immunoblotting (A). Purity of the *ca.* 60-kDa gel-excised candidate protein band was assessed on-gel (B).

The encouraging results in [1] prompted us to determine purity of the candidate target proteins of artemisinin by the reverse-staining method [5], [6], [7], [8]. As a procedure, we incubated 100 μM of probe **5** (Fig. 1) directly into the parasite lysate for 5 min.^3^ Labeled proteins were immobilized by streptavidin-tagged resins and subsequently released in Laemmli׳s sample buffer. Following SDS-PAGE of the protein samples, the electrophoresis gel was reverse stained using a reverse-staining kit (Bio-Rad). The on-gel detected protein bands were excised, destained and crushed in Laemmli׳s SDS-PAGE running buffer. After elution by vigorous agitation, the filtered protein samples were concentrated by ultracentrifugation, and then subjected to SDS-PAGE in duplicate (Fig. 2). The first gel underwent usual Western blotting procedures and was visualized by enhanced chemiluminescence. As a result (Fig. 2A), the molecular size of the isolated single band in lane 3 corresponded effectively to the *ca.* 60-kDa band in the control lane 1, while both low-abundance low molecular-sized candidate target proteins (40- and 39-kDa) were almost undetected in lane 2. Next, the second gel was reverse stained, thereby allowing on-gel detection. As a result (Fig. 2B), a single band of the *ca.* 60-kDa candidate target protein could be visualized without any contaminant.

## Footnotes

3 While in the associated research article [1], we incubated probe **5** with the parasite for 3 h for allowing its covalent binding to the parasite target proteins, in this data article we incubated probe **5** directly into the parasite lysate only for 5 min for allowing the same binding process to occur. This difference in incubation time could be explained by the fact that, in the research article [1], the incubation of the probe was performed in the whole parasite cell culture, thus requiring the molecular probe to first cross the parasite membrane (during uptake of the probe by the parasite) from the culture medium into the parasite intracellular milieu, and then navigate in the parasite cytosol or probably from the cytosol into a specific organelle in order to reach the target proteins for covalent binding. We assumed this process would require a significant amount of time for completion; thereby we allocated a maximum of 3 h incubation in [1]. In this data article, probe **5** was incubated directly into the parasite lysate, which is a cocktail of soluble lysed cell contents or biomolecules. Therefore, the target proteins were readily accessible to the probe for covalent binding and we assumed this could be completed in a significantly reduced amount of time, *i.e.*, 5 min.
